# The predicting role of serum tumor-specific growth factor for prognosis of esophageal squamous cell carcinoma

**DOI:** 10.1186/s12885-023-11602-x

**Published:** 2023-11-06

**Authors:** Xiaoqin Xu, Weigang Wang, Baoguo Tian, Xiaofang Zhang, Yanfen Ji, Jiexian Jing

**Affiliations:** Department of Clinical Laboratory, Shanxi Province Cancer Hospital/Shanxi Hospital Affiliated to Cancer Hospital, Chinese Academy of Medical Sciences/Cancer Hospital Affiliated to Shanxi Medical University, Taiyuan, Shanxi People’s Republic of China

**Keywords:** Esophageal squamous cell carcinoma, Tumor-specific growth factor, Prognosis, Marker

## Abstract

**Objective:**

Tumor-specific growth factor (TSGF) is an immune-related factor that offers good performance in the clinical management of human cancers. However, the role of serum TSGF in esophageal squamous cell carcinoma (ESCC) has not been fully clarified.

**Methods:**

A total of 562 ESCC cases were collected in our study, with available information on preoperative serum levels of TSGF at diagnosis. Preoperative serum TSGF was detected using the rate method. We retrospectively analyzed its correlation with clinicopathological features of ESCC and survival.

**Results:**

The cut-off value of serum TSGF was determined to be 60.5 U/mL by receiver operating characteristic (ROC) analysis. Serum TSGF was associated with gender (*P* < 0.001), tumor location (*P* = 0.022), tobacco use (*P* < 0.001), alcohol consumption (*P* < 0.001), lymph node involvement (*P* = 0.007), and TNM staging (*P* = 0.004). The survival analysis revealed that ESCC patients with high levels of serum TSGF had poorer prognosis than those with high TSGF (*P* = 0.006), especially for male ESCC cases (*P* = 0.001), under 60 year (*P* = 0.036), male middle location (*P* = 0.023), tobacco consumption (*P* = 0.004), G1 + G2 (*P* = 0.031), advanced T staging (*P* = 0.033), lymph node involvement (*P* = 0.003), TNM staging (*P* = 0.003). Univariate Cox regression analysis indicated that exposure to smoking and drinking, tumor grade, T staging, lymph node metastasis, TNM staging, and serum TSGF level were the prognosis-related factors of ESCC. Multivariate regression analysis revealed that smoking history, higher serum TSGF levels, and advanced T stage enhanced the risk of ESCC-related death.

**Conclusion:**

In brief, serum TSGF levels had in relation to malignant features of ESCC. It was positively correlated with survival but was identified as an independent risk factor for ESCC.

## Introduction

Esophageal squamous cell carcinoma (ESCC) is one of the most common malignancies involving the digestive system, and the leading cause of cancer-related deaths worldwide owing to its aggressiveness and adverse survival probability. In China, ESCC is the principal subtype of esophageal cancer and remains the sixth leading cause of cancer incidence and the fourth leading cause of cancer mortality [1]. In recent years, the clinical approaches to overcoming ESCC have focused on the combination therapy comprising operative treatment, chemotherapy, and radiotherapy based on patient background and TNM staging by the American Joint Committee on Cancer (AJCC)/International Union Against Cancer (UICC) clinical stage [2]. Currently, emerging immunotherapy is an additional treatment method [3]. However, tumor development dominated by diverse tumor environments and complex heterogeneity. Despite the more extensive efforts to defeat ESCC and improve the prognosis, it is indispensable to find a reliable clinically beneficial biomarker to trace the progression of ESCC.

Tumor-specific growth factor (TSGF) is a collective name that includes multiple substances related to the malignant growth of tumors. Based on previous efforts, accumulating studies have revealed the importance of serum TSGF for the clinical identification and assessment of treatment effects in various malignancies, such as breast cancer, and digestive tract carcinoma [4–17]. Of note, combining TSGF and conventional biomarkers improved essential clinical utility. The combination of color Doppler ultrasound with serum carcinoembryonic antigen (CEA), carbohydrate antigen 153 (CA15-3), and TSGF detection enhanced the diagnostic efficiency of breast cancer [4]. TSGF and other tumor markers combined with 18 F-FDG-PET achieved approximately 97.3% of diagnostic accuracy, and yielded 100% specificity for discriminating prostate cancer [11]. The joint detection of serum CEA, carbohydrate antigen 72-4 (CA72-4), carbohydrate antigen 19-9 (CA19-9), and TSGF has shown robust discriminatory power in gastric cancer (AUC = 0.913, sensitivity: 88.9%) [6]. Similarly, the combined assay with serum TSGF, carbohydrate antigen 242 (CA242), and CA19-9 appeared to be able to identify pancreatic cancer [7]. Meanwhile, three serum markers, consisting of AC007271.3, squamous cell carcinoma antigen (SCCA), and TSGF, resulted in an AUC of 0.917 with a sensitivity of 80.0% and specificity of 93.1% in oral squamous cell carcinoma [14]. TSGF predicts the survival consequence of pancreatic cancer patients undergoing cryoablation treatment [8], utilizes to monitor the treatment response in colon cancer [9], and determines the survival of bladder cancer patients undergoing robot-assisted radical cystectomy [10]. For primary hepatocellular carcinoma, patients carrying increased TSGF levels obtained a reduced 3-year survival rate, showing its value for evaluating survival benefit of transcatheter arterial chemoembolization (TACE) [12]. For osteosarcoma, higher TSGF levels decreased 5-year survival rate [15]. Yang L et al. performed a study with 753 retecal carcinomas and demonstrated that decline of TSGF in 5th postoperative day (POD) less than 10 U/mL showed a positive correlation with anastomotic leakage (AL) after anterior resection for rectal cancer with a double stapling technique [16]. Altogether, these results demonstrate that serum TSGF plays a crucial role in the screening, prognosis prediction, and treatment outcome of cancer. However, its role in ESCC is unclear.

Unexpectedly, only a few studies are currently available that have examined the role of TSGF in esophageal carcinoma. The combined detection of AgNOR, SCCA, CEA, and TSGF was favorable for screening esophageal carcinoma [18]. Therefore, we retrospectively reviewed 562 ESCC cases to assess their clinicopathological relevance and potential role as prognostic indicators for ESCC.

## Materials and methods

### Patients

Altogether, 562 cases with ESCC were collected at Shanxi Province Cancer Hospital from January 2016 to July 2018. None of the subjects received neoadjuvant chemoradiotherapy before operative treatment. All subjects undertake surgery and ESCC diagnosis was consequently confirmed by pathologists. Clinical staging of ESCC was dependent on the 8th edition of the esophageal cancer TNM staging system issued by the American Joint Commission on Cancer and Union for International Cancer Control. Our present study was conducted following the World Medical Association Declaration of Helsinki.

Included criteria:

1. Patients with first hospitalization from January 2016 to July 2018;
2. All cases had complete medical records;
3. Patients who did not receive pre-surgery neoadjuvant chemoradiotherapy for ESCC;
4. Patients who underwent the esophageal resection;
5. The final diagnosis of ESCC was confirmed by pathologists.

Excluded criteria:

1. Patients who received pre-surgery neoadjuvant chemoradiotherapy for ESCC;
2. Patients who did not conduct the esophageal resection;
3. Patients who had incomplete medical material;
4. Patients with unconfirmed pathological diagnosis.

### Data collection

The clinical data were obtained from patient’s medical records and included basic information such as age at diagnosis and sex; cancer-related information such as tumor location, tumor grade, pathologic T stage, lymph node status, and TNM stage; lifestyle information such as tobacco consumption and alcohol consumption; preoperative detection based on blood assays including serum TSGF level; and treatment options. Follow-up was performed via telephone and regular outpatient consultation according to National Comprehensive Cancer Network (NCCN) guidelines. Overall survival (OS) time was calculated from the date of surgery to the date of the most recent follow-up or death. And the follow-up period ended in December 2022.

### Serum TSGF assay

A 3-mL sample of peripheral blood was withdrawn from ESCC patients and the serum was obtained for detection after centrifugation at 1000 × g for 10 min. Hemolysis, jaundice, and lipids were excluded from all samples. Importantly, if the serum TSGF was not detected immediately, it was stored at -20℃ or -80℃ and protected from repeated freezing and thawing. Serum TSGF levels were measured by BIOELAB ES-200 Automatic Biochemistry Analyzer(Changsha, China) according to the manufacturer’s instructions(tumor-specific growth factor assay kit, Hunan Newland Biotechnology Co., Ltd, China). After the completion of the test, the results were obtained based on the standard curve.

### Statistical analysis

All data were processed using SPSS 22.0 software (IBS SPSS, Armonk, NY, USA). The ideal cut-off values for TSGF levels were determined using receiver operating characteristic (ROC) analysis: the survival status of ESCC patients as the status variable and TSGF as the test variable. Its correlation with clinicopathological features of ESCC was investigated via the chi-square test. Overall survival and stratified analysis were examined using the Kaplan-Meier method and log-rank test. The univariate and multivariate analyses were executed following the Cox proportional hazards regression model. P values less than 0.05 were regarded as statistically significant.

## Results

### The characteristics of ESCC patients

As shown in Tables 1, 562 ESCC patients were recruited including 372 males and 190 females. The age of the enrolled ESCC cases ranged from 40 years to 81 years old, with a median age of 61 years. Drinking and smoking habits were risk factors promoting ESCC. In our study, 286 cases with ESCC had exposure to tobacco use, and 181 patients harbored an alcohol-drinking habit. The dominant location was the middle esophagus in 417 cases, the lower segment in 116 cases, and the upper esophagus in only 29 cases. For tumor grade, moderately differentiated ESCC is the dominant subgroup, accounting for the majority of the total cases (62.46%), followed by poorly differentiated (35.77%) and well-differentiated ESCC (1.78%). Moreover, 66.01% of ESCC patients were at the advanced T stage at diagnosis, and 33.99% were diagnosed at the early T stage. In total, 42.53% of ESCC patients harbored advanced TNM stages at the time of diagnosis, and 43.77% had lymph node involvement. Of note, the patients at the II stage were the majority of all ESCC cases (266/562), followed by III stage (207/562).

**Table 1:** The clinical characteristics of ESCC patients

Characteristics	n
**Gender**	
Male/Female	372/190
**Age**	
< 60 years/≥60 years	232/330
**Location**	
upper/middle/lower	29/417/116
**Smoking history**	
No/Yes	276/286
**Drinking history**	
No/Yes	381/181
**Grade**	
G1/G2/G3	10/351/201
**TNM staging**	
I/II/III/IV	57/266/207/32
**T stage**	
T1/T2/T3/T4	63/128/369/2
**Lymph node status**	
Negative/Positive	316/246

### The relationship between serum TSGF and the features of ESCC

We determined the cut-off point of serum TSGF as 60.5 U/mL by ROC analysis(AUC = 0.554, *P* = 0.028), and then all collected cases were separated into two groups at this threshold: a high TSGF group and a low TSGF group. Our analysis revealed that increased serum TSGF was related to sex (*χ2* = 23.351, *P* < 0.001), tumor location (*χ2* = 7.654, *P* = 0.022), smoking history (*χ2* = 14.898, *P* < 0.001), drinking history (*χ2* = 13.632, *P* < 0.001), lymph node involvement (*χ2* = 7.199, *P* = 0.007), and TNM staging (*χ2* = 8.233, *P* = 0.004) (Table 2). However, there was no statistical correlation between serum TSGF level and age at diagnosis (*P* > 0.05), tumor differentiation (*P* > 0.05), or pathologic T stage (*P* > 0.05). These findings implied that elevated TSGF may be a promising serum biomarker concerning the malignant property of ESCC.

**Table 2:** The relationship between serum TSGF level and clinicopathological parameters in patients with ESCC

	TSGF		*χ2*	*P*
	low(n = 319)	high(n = 243)		
**Gender**				
Male	238(64.0)	134(36.0)	23.351	< 0.001***
Female	81(42.6)	109(57.4)		
**Age**				
< 60 years	138(59.5)	94(40.5)	1.192	0.275
≥ 60 years	181(54.8)	149(45.2)		
**Location**				
upper	23(79.3)	6(20.7)	7.654	0.022*
middle	237(56.8)	180(43.2)		
lower	59(50.9)	57(49.1)		
**Smoking history**				
No	134(48.6)	142(51.4)	14.898	< 0.001***
Yes	185(64.7)	101(35.3)		
**Drinking history**				
No	196(51.4)	185(48.6)	13.632	< 0.001***
Yes	123(68.0)	58(32.0)		
**Grade**				
G1 + G2	207(57.3)	154(42.7)	0.138	0.710
G3	112(55.7)	89(44.3)		
**Lymph node stasus**				
Neagative	195(61.7)	121(38.3)	7.199	0.007**
Positive	124(50.4)	122(49.6)		
**T stage**				
T1 + T2	119(62.3)	72(37.7)	3.621	0.057
T3 + T4	200(53.9)	171(46.1)		
**TNM staging**				
I+II	200(61.9)	123(38.1)	8.233	0.004**
III+IV	119(49.8)	120(50.2)		

*: *P* < 0.05, **: *P* < 0.01, ***: *P* < 0.001

### Serum TSGF predicts survival for prognosis of ESCC patients

**Fig. 1:**
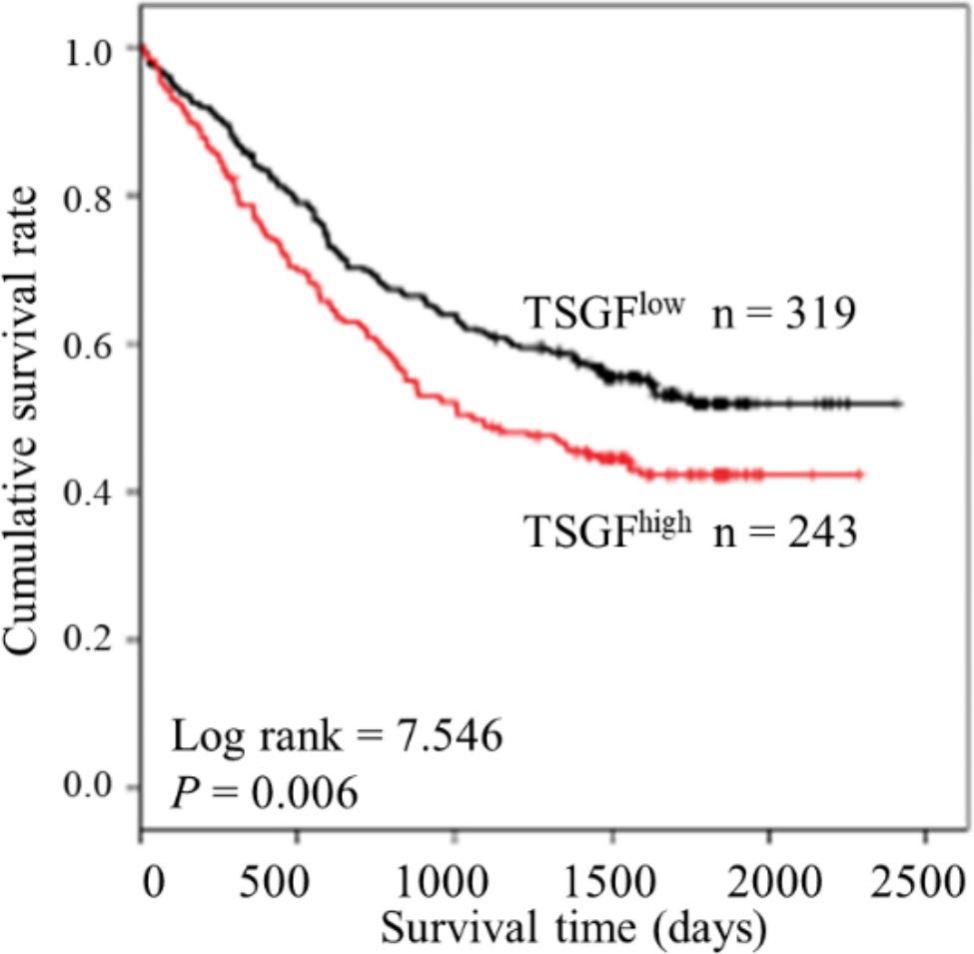
The prognostic predictive role of preoperative serum TSGF in ESCC

Given its predictive role for cancer-related prognosis, we further performed the survival analysis. We found that ESCC patients with low levels of serum TSGF had worse clinical outcomes than those with high levels of TSGF (*P* = 0.006) (Fig. 1). This result suggested that serum TSGF may be a prognostic indicator of ESCC.

**Fig. 2:**
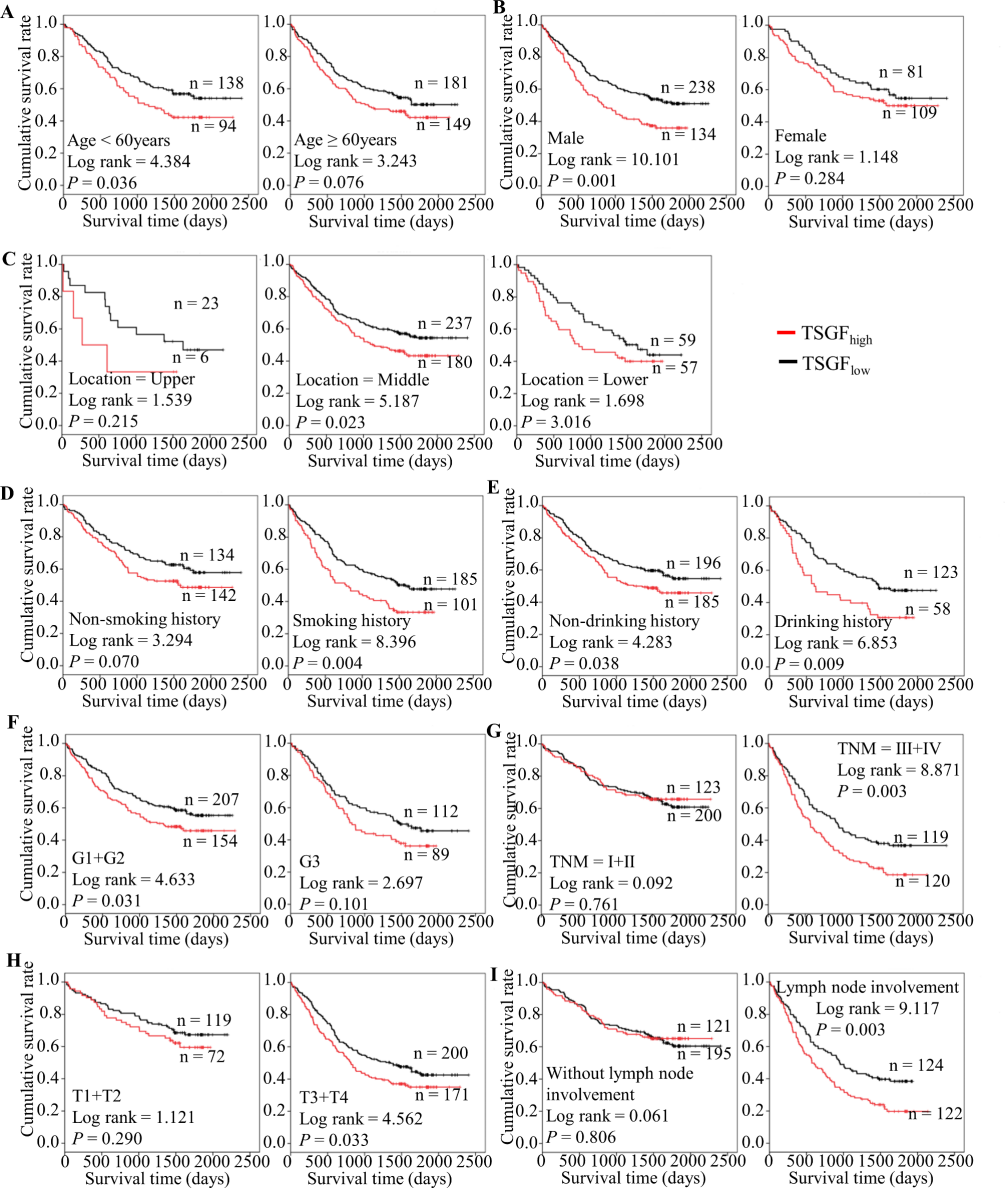
Predicating role of preoperative serum TSGF level for the OS of ESCC patients. **A**-**I**: Kaplan-Meier survival curves of ESCC patients with different TSGF levels combined with diverse features such as age (**A**), sex (**B**), tumor location (**C**), smoking history (**D**), drinking history (**E**), tumor grade (**F**), TNM staging (**G**), T staging (**H**) and lymph node status (**I**)

**Fig. 3:**
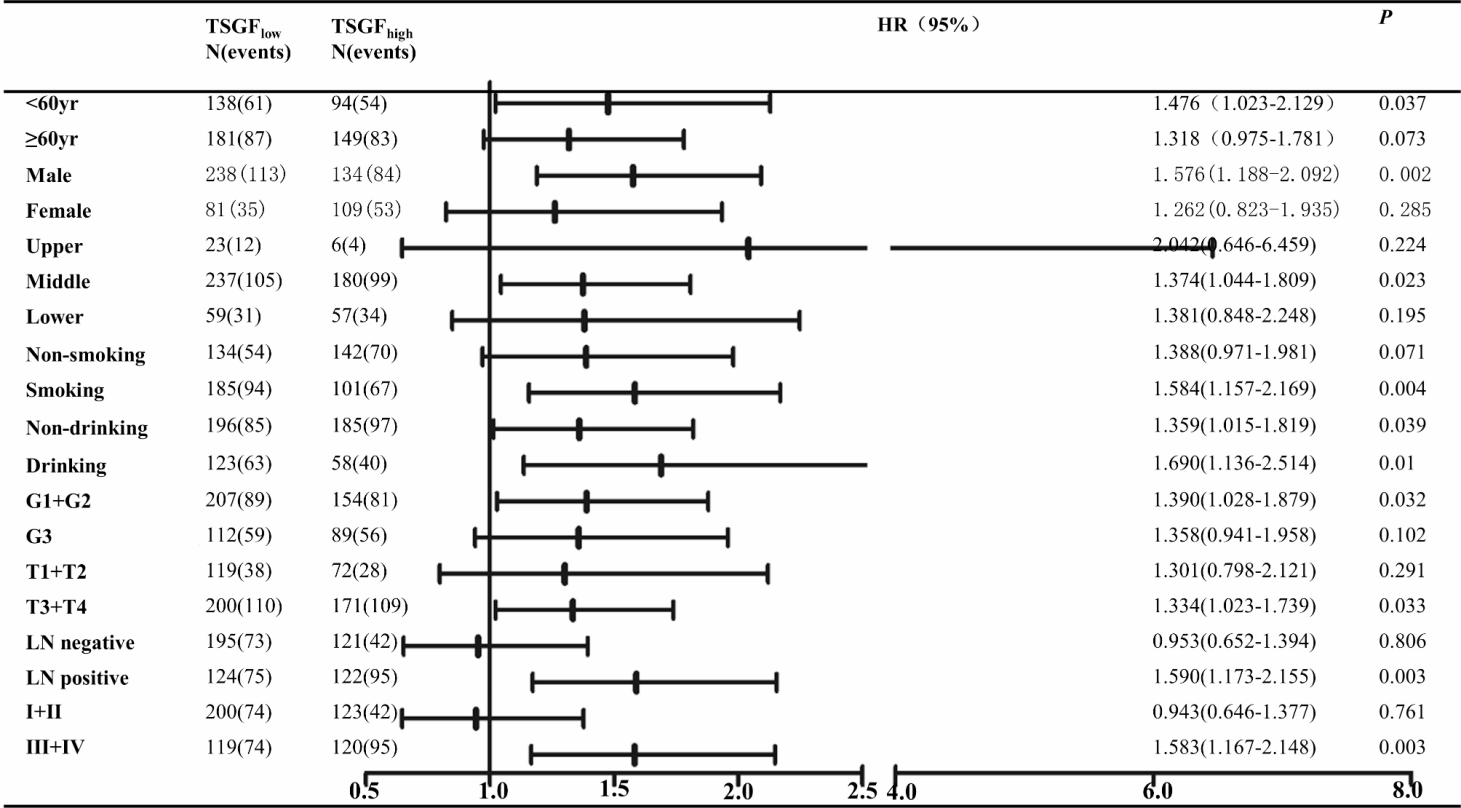
Predicating role of preoperative serum TSGF level for risk of deaths of ESCC patients. LN: lymph node

To tailor the refined prediction, we further performed stratified analysis. The results indicated that increased serum TSGF was an excellent predictor for adverse outcomes in ESCC patients with younger age (< 60 years) (*P* = 0.036), male sex (*P* = 0.001), middle location (*P* = 0.023), tobacco consumption (*P* = 0.004), G1 + G2 (*P* = 0.031), advanced T staging (*P* = 0.033), lymph node involvement (*P* = 0.003), and TNM staging (*P* = 0.003). Of note, we found that higher serum TSGF levels in ESCC patients with or without a drinking history were prone to shorten the survival time. (Fig. 2). Additionally, we evaluated the predictive role of different levels of TSGF for ESCC-related deaths(Fig. 3). The analysis showed that high TSGF increased 1.476-fold the risk of death for ESCC under 60 year group than level TSGF. Similarly, high TSGF was a risk factor for poor prognosis in the male ESCC group(HR = 1.576). In another subgroup, for the cases with middle ESCC, having smoking and drinking habits, well and moderate differentiation, positive lymph node, and advanced TNM staging, high TSGF acted as an unfavorable factor for survival of ESCC.

### Serum TSGF level is an independent predictor of ESCC prognosis

To further weigh the role of serum TSGF level in survival prediction in ESCC patients, we then indicated that tobacco consumption (*P* = 0.005), drinking history (*P* = 0.043), grade (*P* = 0.040), pathologic T stage (*P* < 0.001), lymph node metastasis (*P* < 0.001), TNM staging (*P* < 0.001), and serum TSGF level (*P* = 0.006) may lead to unfavorable clinical outcomes in patients with ESCC (Table 3). The multivariate Cox regression analysis revealed that smoking history (*HR*: 1.423, *95%CI*: 1.06–1.91, *P* = 0.019), higher serum TSGF levels (*HR*: 1.330, *95%CI*: 1.048–1.688, *P* = 0.019), and advanced pathologic T stage (*HR*: 1.771, *95%CI*: 1.332–2.355, *P* < 0.001) were independent prognostic predictors for the survival of ESCC patients.

**Table 3:** The univariate and multivariate analysis

	univariate					multivariate			
	*HR*	*P*	*95%CI*			*HR*	*P*		*95%CI*
**Age**	1.112	0.379	0.878–1.409						
**Gender**	0.803	0.088	0.625–1.033						
**Location**									
Upper		0.446							
Middle	0.837	0.495	0.503–1.393						
Lower	0.987	0.963	0.571–1.706						
**Grade**	1.282	0.040	1.012–1.625			1.082		0.522	0.850–1.377
**T stage**	2.157	< 0.001	1.637–2.843			1.771		< 0.001	1.332–2.355
**Smoking history**	1.394	0.005	1.103–1.763			1.423		0.019	1.060–1.910
**Drinking history**	1.283	0.043	1.007–1.634			1.000		0.999	0.737–1.358
**Lymphatic metastasis node status**	2.661	< 0.001	2.097–3.376			1.326		0.495	0.590–2.982
**TNM stage**	2.814	< 0.001	2.218–3.570			1.910		0.120	0.845–4.318
**TSGF**	1.383	0.006	1.096–1.746			1.330		0.019	1.048–1.688

Note: Age(< years vs. ≥ years), gender: female vs. male, location: middle vs. upper, lower vs. upper, grade: G3 vs. G1 + G2, T stage: T3 + T4 vs. T1 + T2, smoking history: yes vs. no, dringking history: yes vs. no, lymph node status: positive vs. negative, TNM stage: III+IV vs. I+II, TSGF: high vs. low

## Discussion

In our present research, we investigated the predictive property of preoperative serum TSGF and explored its clinical utility in ESCC. We analyzed the correlation between serum TSGF and some clinical parameters, including the survival of ESCC. We found that it correlated with sex, tumor location, smoking and drinking history, TNM stage, and lymph node involvement.

Epidemiologically, smoking and alcohol drinking enhanced the risk of ESCC [19]. Even the studies depicted tobacco and alcohol may play a positive synergistic effect to increase 3-fold of the risk of suffering ESCC compared with the predicted risk of any single factor alone(only 20–30%) [20]. In Non-small cell lung cancer, the median and positive rates of TSGF were 64(56–67)μ/mL and 10.14%(28/276). However, TSGF level showed no statistical difference between smokers(median: 63, range: 53–66) and non-smokers(median: 62, range: 51–67) (P > 0.05) [21]. To date, there was no report about its relationship with drinking alcohol. We firstly indicated that TSGF was associated with smoking and drinking history. And higher TSGF in the smoking and alcohol-drinking groups predicted poor clinical outcome of ESCC. More importantly, habitually consuming tobacco, alcohol, and areca nuts can influence the age-onset of male ESCC [22].

Commonly, metastasis leads to an unfavorable clinical outcome of ESCC. For ESCC, lymph metastasis showed a remarkable correlation with malignant features such as tumor differentiation, perineural invasion, advanced-stage tumor, and venous invasion. It predicted a worse prognosis and could act as an independent prognostic factor for ESCC [23]. In our cohort, 246 of 302(43.77%) ESCC cases harbored lymph node involvement, which was slightly higher than the previous report with 36.8% lymph node metastasis [23]. Min BH et al. demonstrated that the different LNM rates may be associated with tumor invasion depth [24]. The LNM rates of tumors invading the lamina propria, muscularis mucosa, and SM1 layers were 3.7%, 15.5%, and 40.7%, respectively. To date, there have been few available studies on the predictive role of the serum level of TSGF for lymph node involvement in cancer. TSGF is a polypeptide that triggers initiation and facilitates metastasis of cancer. TSGF levels have been related to tumor differentiation in pancreatic cancer [8] and lymph node involvement in colon cancer [9]. Our previous study revealed that increased serum TSGF may enhance the emergence of multifocality and the development of PTC, suggesting that TSGF may be qualified as an excellent promising marker for predicting lymphatic involvement in PTC [25]. Our results focused on the clinical utility of TSGF in cancer. Nevertheless, its role in ESCC for predicting prognosis was not uncovered. Our analysis supported that increased TSGF was likely to develop metastasis involving the lymph node in ESCC. This result suggested that serum TSGF may play a distinct role in cancer progression.

In general, the TNM stage represents the tumor burden in tumor-bearing patients. Advanced TNM stage not only results in increased metastasis probability, including more lymph node involvement, but also contributes to poor survival. In the past several years, the therapeutic benefit of ESCC patients was limited by the late diagnosis and asymptomatic early disease [1]. In our present study, we identified that the cases were predominantly at stage II, and 239 of 562 (41.72%) had advanced disease. Moreover, the ESCC patients with high TSGF levels were prone to be at advanced TNM stages. And TNM stage was a predictor of lymph node involvement and poor prognosis [26]. Our result was indicative of its pro-tumor effect in ESCC progression. In addition, there was no significant correlation with other traits, such as age, tumor grade, or pathologic T stage.

To date, whether serum TSGF has a connection with cancer-related deaths has been previously described in several types of cancer. It has been reported that serum TSGF exhibits a superior early detection capability [4–7, 11, 13], prognosis prediction role [12, 15], and response to the therapeutic outcomes [8–10, 12, 14–16] of malignancies. Based on the previous exploration, combining serum TSGF with traditional biomarkers or other detection tools caused better diagnostic accuracy [5]. Higher levels appeared to correlated with worse clinical outcomes of cancer [12, 15]. Our findings indicated that ESCC patients with decreased serum TSGF had a better prognosis than those with high TSGF. This finding seemed to be consistent with the predictive value of hepatocellular carcinoma [12] and osteosarcoma [15]. Furthermore, we conducted the strata analysis to achieve the satisfactory survival prediction for the special ESCC patients. We found that younger-aged ESCC patients with elevated serum TSGF had worse survival. There were similar results in the ESCC subgroup with male cases, middle esophagus, tobacco and alcohol consumption, well and moderate differentiation, advanced TNM staging and pathologic T stage, and lymph node metastasis. These findings might aid clinicians in determining the population at risk for exposure for precise therapeutic intervention and clinical management.

Finally, the univariate analysis showed that higher serum TSGF levels, some malignant features including TNM stage, lymph node status, pathologic T stage, and ESCC lifestyle factors such as tobacco consumption and alcohol exposure could lead to unfavorable clinical outcomes in patients with ESCC. However, multivariate analysis showed that only advanced pathologic T stage, smoking habit, and increased serum TSGF increased the risk of ESCC-related prognosis. Our results suggested it’s necessary to explore the clinical implications of serum TSGF in ESCC.

There were also some limitations in this study. Noticeably, the cases at stage II accounted for most of our cohorts, while the minority were in stage IV. In our cohort, not all subjects had received the same post-operation treatment, and due to the relatively small sample size, it was unfavorable to perform a subgroup analysis based on different treatment options. In addition, the sample size from the single center was limited, although it was larger than the previous cohort. Therefore, it’s necessary to enlarge the sample from multiple centers to obtain a better performance of TSGF.

## Conclusion

Collectively, serum TSGF was relevant to malignant features of ESCC. Increased TSGF predicted the adverse prognosis of ESCC, especially for male patients aged under 60 and with lymph node involvement, advanced disease stage, and smoking and drinking habits. Moreover, the serum TSGF was qualified as an independent predictor for the clinical outcome of ESCC by multivariate analysis. Since the assay of cytokine levels in ESCC patients is feasible and convenient in clinical practice, we will perform further observation to investigate its clinical utility and the molecular mechanisms underlying TSGF activity to shed light on its role in ESCC progression.

## Data Availability

The data used to support the findings of this study are available from the corresponding author upon reasonable request.
